# Additive and interaction effects of working memory and motor sequence training on brain functional connectivity

**DOI:** 10.1038/s41598-021-02492-9

**Published:** 2021-11-29

**Authors:** Priska Zuber, Laura Gaetano, Alessandra Griffa, Manuel Huerbin, Ludovico Pedullà, Laura Bonzano, Anna Altermatt, Charidimos Tsagkas, Katrin Parmar, Patric Hagmann, Jens Wuerfel, Ludwig Kappos, Till Sprenger, Olaf Sporns, Stefano Magon

**Affiliations:** 1 Division of Cognitive Neuroscience, Faculty of Psychology, University of Basel, Basel, Switzerland; 2 F. Hoffmann-La Roche Ltd., Basel, Switzerland; 3 Department of Clinical Neurosciences, Division of Neurology, Geneva University Hospitals and Faculty of Medicine, University of Geneva, Geneva, Switzerland; 4 Center of Neuroprosthetics, Institute of Bioengineering, École Polytechnique Fédérale De Lausanne (EPFL), Geneva, Switzerland; 5 Medical Image Analysis Center (MIAC AG), Basel, Switzerland; 6 Department of Experimental Medicine, Section of Human Physiology, University of Genoa, Genoa, Italy; 7 Italian Multiple Sclerosis Foundation, Scientific Research Area, Genoa, Italy; 8 Department of Neuroscience, Rehabilitation, Ophthalmology, Genetics, Maternal and Child Health, University of Genoa, Genoa, Italy; 9 Neurologic Clinic and Policlinic, Departments of Medicine, Clinical Research and Biomedical Engineering, University Hospital Basel and University of Basel, Basel, Switzerland; 10 Translational Imaging in Neurology (ThINk) Basel, Department of Medicine and Biomedical Engineering, University Hospital Basel and University of Basel, Basel, Switzerland; 11 Reha Rheinfelden, Rheinfelden, Switzerland; 12 Department of Radiology, Centre Hospitalier Universitaire Vaudois (CHUV) and University of Lausanne (UNIL), Lausanne, Switzerland; 13 Department of Biomedical Engineering, University of Basel, Basel, Switzerland; 14 Research Center for Clinical Neuroimmunology and Neuroscience Basel (RC2NB), Departments of Medicine, Clinical Research and Biomedical Engineering, University Hospital Basel and University of Basel, Basel, Switzerland; 15 Department of Neurology, DKD Helios Klinik, Wiesbaden, Germany; 16 Department of Psychological and Brain Sciences, Indiana University, Bloomington, IN USA; 17 Indiana University Network Science Institute, Indiana University, Bloomington, IN USA; 18 Roche Pharma Research and Early Development, Roche Innovation Center Basel, F. Hoffmann-La Roche Ltd., Basel, Switzerland

**Keywords:** Working memory, Cognitive neuroscience, Learning and memory, Motor control

## Abstract

Although shared behavioral and neural mechanisms between working memory (WM) and motor sequence learning (MSL) have been suggested, the additive and interactive effects of training have not been studied. This study aimed at investigating changes in brain functional connectivity (FC) induced by sequential (WM + MSL and MSL + WM) and combined (WM × MSL) training programs. 54 healthy subjects (27 women; mean age: 30.2 ± 8.6 years) allocated to three training groups underwent twenty-four 40-min training sessions over 6 weeks and four cognitive assessments including functional MRI. A double-baseline approach was applied to account for practice effects. Test performances were compared using linear mixed-effects models and t-tests. Resting state fMRI data were analysed using FSL. Processing speed, verbal WM and manual dexterity increased following training in all groups. MSL + WM training led to additive effects in processing speed and verbal WM. Increased FC was found after training in a network including the right angular gyrus, left superior temporal sulcus, right superior parietal gyrus, bilateral middle temporal gyri and left precentral gyrus. No difference in FC was found between double baselines. Results indicate distinct patterns of resting state FC modulation related to sequential and combined WM and MSL training suggesting a relevance of the order of training performance. These observations could provide new insight for the planning of effective training/rehabilitation.

## Introduction

Motor learning describes the human ability to acquire new motor skills^1^ and has been defined as “[…] increased spatial and temporal accuracy of movement with practice.”^2^. Learning new motor skills on the one hand results from explicit learning, where the learner relies on his declarative memory by applying performance rules in order to acquire and control a new set of movements. On the other hand, acquiring new skills can also occur implicitly through repetition of movements with practice based on visual or tactile sensory feedback, released from explicit memory control^3^. Supporting a distinction in explicit and implicit learning of movements, it has been demonstrated that, after damage of the medial temporal lobe due to brain injury or stroke, the capacity of implicit motor sequence learning (MSL) can be retained, whereas the capacity of explicit learning of motor skills is impaired^4^. Therefore, MSL could be an interesting function to target in the context of training and rehabilitation, since it has been hypothesized that MSL is not a purely motor function and that cognitive functions such as working memory are involved already in early stages of MSL^2,5^.

Working memory (WM) refers to a cognitive system which temporarily maintains, stores and manipulates information and acts as an interface between perception, long-term memory and action^6,7^. Following Baddeley’s multicomponent model, WM can be divided into a visuospatial, a verbal, a coordinative and a buffer component^7^. Since WM is centrally involved in numerous higher order cognitive functions^8^ and plays a role in age-related cognitive decline^9^, WM training has been suggested to improve WM capacity, suggesting that it may ameliorate cognitive decline^10^. WM trainings gained huge interest over the last decade and numerous studies showed their efficacy in improving WM capacity as well as transfer effects to cognitive domains that are not specifically targeted by the training^11,12^.

There is evidence that MSL is related to WM abilities. It has been shown that the spatial component of WM and visuomotor adaptation share common processes^5^ and the individual spatial WM performance predicts the rate of implicit MSL^13^. Furthermore, a correlation between visual and visuospatial WM with reaction time change in a serial reaction time task has been described which further supports a sharing component between WM and implicit MSL^14^. These shared components are also mirrored in the neural activity. Functional magnetic resonance imaging (fMRI) evidence indicates that brain regions conventionally associated with motor activation show sub-regions that contribute to motor and cognitive processes jointly^15^. To that end, the right dorsolateral prefrontal cortex, parietal and premotor regions, basal ganglia as well as cerebellar areas have been described to be involved in MSL as well as WM performance^13,15^. Regarding brain functional connectivity (FC), dynamic changes in terms of integration within and between the premotor and sensorimotor network have been shown to be associated with MSL. Moreover, it has been shown that this functional integration decreases with practice over a 4-week training of motor learning^16^. Regarding WM and motor training studies, a meta-analysis described that across 53 studies showing brain activation decreases in numerous brain regions solely the dorsolateral prefrontal cortex showed a consistent activation decrease. Additionally, training-related increases were consistent in the salience-network (supplementary motor area, anterior cingulate cortex, and inferior frontal gyrus, anterior insula), dorsal attention network (the superior parietal cortex, intraparietal sulcus, frontal eyefield), striatum, thalamus, ventral and dorsal visual and superior temporal cortices^17^. Whereas these regions showed FC changes during task-fMRI, recent studies further indicate changes in resting state FC following WM training^18^ but also after motor training^19^.

Despite the reported relationship between WM and MSL, to our knowledge there are currently no studies that investigate the interactive effects of MSL and WM training on brain activation patterns or behavioral outcomes. It is, therefore, necessary to investigate the sequential order of training administration, in order to study the interactive neural and behavioral effects of MSL and WM training. Thus, the present study aimed to investigate how brain FC and behavioural outcomes are modulated by sequential training programs with varying order of administration (WM + MSL and MSL + WM) or combined administration (WM and MSL in the same session). In the first setting, the purpose was to quantify the additive effects, while, in the second case, the interactive ones.

## Methods

### Participants

Fifty-four healthy subjects were included in the study (27 women and 27 men; mean age: 30.8 ± 8.5, age range: 20–51 years). Inclusion criteria were: age between 18 and 65 years old, right-handedness (above the 5th decile) according to the extended version of the Edinburgh handedness questionnaire^20^ as well as no history of neurological, psychiatric disorders or substances abuse. Written informed consent was obtained from each participant after a detailed explanation of the study procedures. The study was approved by the local ethics committee (Ethikkommission Nordwest und Zentralschweiz) and was conducted in accordance with the Declaration of Helsinki.

### Experimental design

**Figure 1:**
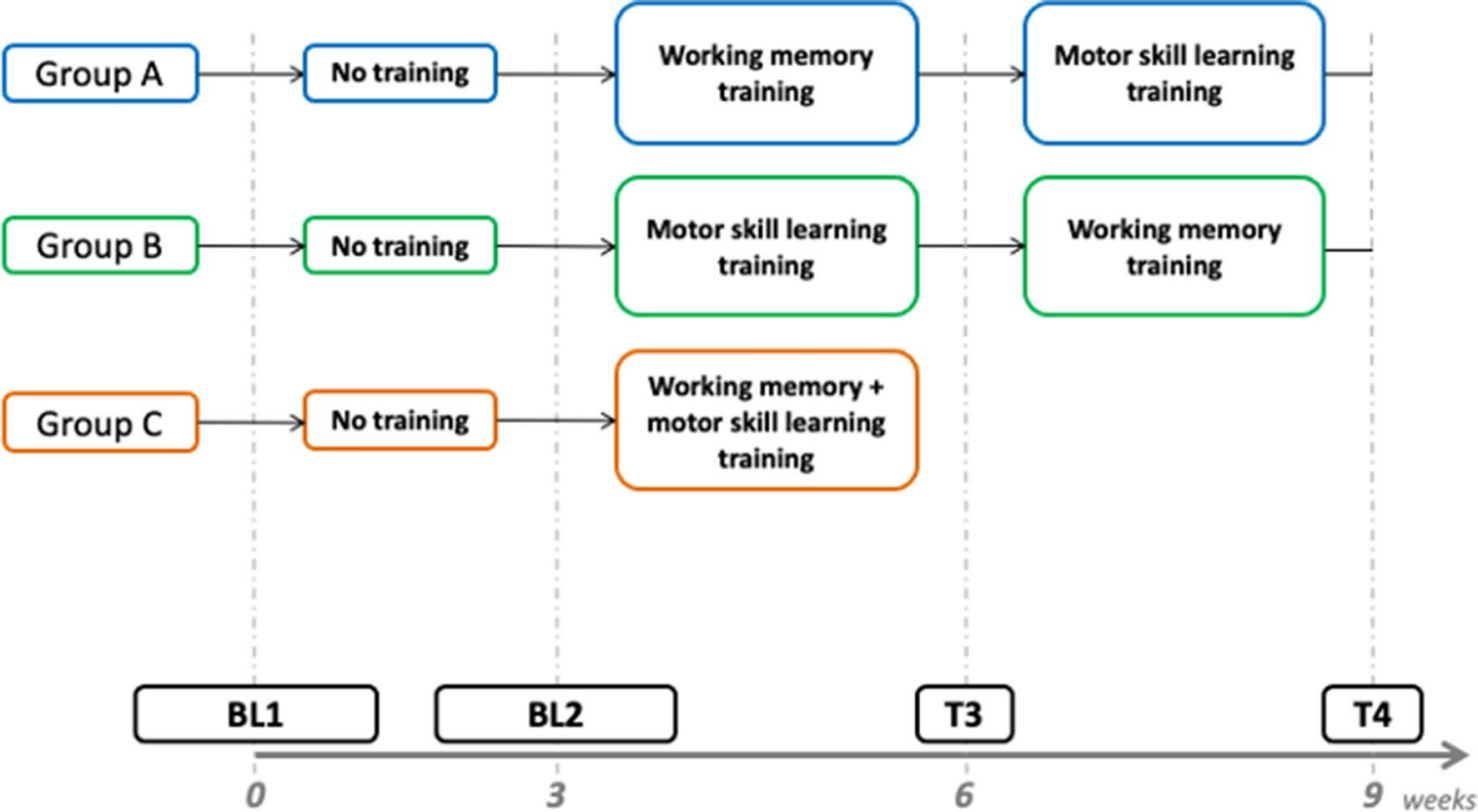
Study procedures. Each assessment (BL 1, BL2, T3 and T4) included an MRI and cognitive testing.

All participants were randomly assigned to three groups (A, B and C) using the minimization approach in order to create comparable groups in terms of age and gender (see Table 1). All groups underwent a WM training (WMT) and a MSL training (MSLT), both consisting of 12 sessions each (four times a week). Each WMT’s session lasted 25 min and the MSLT’s session lasted 15 min. Group A and group B performed the two trainings sequentially within 6 weeks. The second 3-week training started after the completion of the 3 weeks of the first one. The two trainings were performed with a different order for the two groups: WMT + MSLT for group A, and MSLT + WMT for group B. This design allowed to investigate the additive effects of the two trainings. Group C performed both trainings within 3 weeks (for 12 sessions, each with a total duration of 40 min). Therefore, each training session included WMT and MSLT in order to investigate the interaction effects (WMTxMSLT). Additionally, participants underwent a first baseline magnetic resonance imaging (MRI) 3 weeks before the first training session (BL1) and a second baseline MRI within 2 days before the first training session (BL2, Fig. 1). The dual baseline approach has been employed to assess the repetition effect of multiple MRIs on the FC changes as well as neuropsychological test performance. All participants underwent a third MRI within 1 week from the end of the first training program (T3). Groups A and B underwent a fourth MRI after the second training period (T4, within 1 week; Fig. 1).

**Table 1:** Demographic characteristics and training adherence for the three groups.

	Age, years (M ± SD)	Sex (f/m)	MSL training sessions, n (M ± SD)	WM training sessions, n (M ± SD)
Group A	30.1 ± 8.8	9/9	12.2 ± 3.75	10.8 ± 3.0
Group B	30.6 ± 8.4	9/9	13.7 ± 3.29	12.0 ± 2.59
Group C	30.1 ± 8.9	9/9	11.7 ± 3.12	12.2 ± 1.96

M, mean; SD, standard deviation; n, number.

### Training

All training sessions have been performed at home using touch-screen tablet devices provided by the research group. The trainings were developed using Java in Android Studio v1.5.1 (https://developer.android.com/studio/index.html) and downloaded on Lenovo TAB A10 with Android 4.4 as operating system. Both trainings were based on an adaptive design. Therefore, the difficulties of the tasks were modulated according the participant’s performance.

The training for MSL was developed in-house based on a single-paradigm design. The participants learned the procedural knowledge needed to execute a series of actions following a cue presented on the screen. Specifically, four circles were presented on the screen, each one associated with a finger of the dominant hand, from left (index finger) to right (pinkie). The thumb was not included in the sequence. The participants were asked to touch the circle that changed colour with the corresponding finger (supplementary materials, Fig. 1). The levels were based on the number of items included in the sequence (the first level included 5 items) and on the interstimulus interval (ISI), i.e. 1200 ms, 900 ms and 600 ms. For every level, the sequence of items was repeated 10 times. The level of difficulty was increased stepwise, if (a) the accuracy exceeded 80% and (b) reaction time of the correct answers on the last five repetitions was faster compared to the first five repetitions on average. If the participant failed to increase the level of difficulty for three consecutive repetitions of the same level, the difficulty was decreased by one step.

The COGNI-TRAcK^21–23^ was used to train the WM skills (supplementary materials, Fig. 2). Briefly, it includes the following three trainings. (1) The visuospatial training (WMT-VS), in which circles were presented one at a time in a three-by-three grid-like interface. Participants had to remember the location and the order of the stimuli. Levels were defined by the number of stimuli (the first level included four elements) and by the ISI, i.e. 2000 ms, 1500 ms and 1000 ms. The difficulty was increased if the accuracy was 100% for the levels with less than eight stimuli and 80% for higher number of stimuli. (2) Operation N-back training (WMT-OP), in which paired of numbers (from 1 to 4) were showed in a random sequence on the screen (e.g., 3 + 4). Participants were asked to memorize the sum of the two numbers presented (ranging from 2 to 8) and select the correct answer on the screen, which referred to N stimuli ago. The levels were defined by the value of N and by the ISI (from 5000 to 3000 ms in steps of 500 ms). The difficulty was changed if the accuracy was higher than 80%. (3) Dual N-back training (WMT-DT), in which the stimuli (numbers for 1 to 4) were randomly presented one at a time in one of the four possible positions along a line. The participants were asked to memorize the number and the position. Then, they were also asked to select the correct item on screen using the left hand (1, 2, 3 or 4), and the correct position of the stimulus appeared N stimuli ago using the right hand. The definition of the level remained the same as for the previous task. The change of the level occurred if the accuracy was higher than 75%.

### Cognitive assessment

At all four MRIs (BL1, BL2, T3 and T4), an additional cognitive assessment was performed. All participants underwent neuropsychological assessment addressing attention, processing speed and working memory using both paper/pencil and computerized tests. Namely, information processing speed was assessed using the Paced Auditory Serial Addition Task (PASAT^24^) and the oral version of the Symbol Digit Modalities Test (SDMT^25^), verbal WM was assessed using the forward and backward digit span (WAIS-IV^26^) and visuospatial WM was assessed with the Corsi Block Tapping Test^27^ and a visuospatial n-back task. Additionally, finger dexterity was assessed using the nine-hole peg test (9HPT^28^), performed for the right and left hand. The 30-min assessments were performed at the Department of Neurology, University Hospital Basel by trained psychologists.

### MRI protocol

MRI data were collected on a 3 Tesla scanner (Siemens Prisma) using a 64-channel head coil. The MRI protocol included a 3D T1-weighted (T1w; MPRAGE, TR = 2400 ms, TE = 2.32 ms, TI = 1100 ms, voxel, flip angle = 8°, number of slides = 256, voxel size = 0.7 mm^3^ isotropic), and a multi-band accelerated echo-planar imaging (EPI) sequence^29^ used to collect the FC data (TR = 768 ms, TE = 37 ms, flip angle = 52°, bandwidth = 2290 Hz/Px, multi-band accelerator factor = 8, number of slides = 72, voxel size = 2 mm^3^ isotropic, phase-encoding = anterior–posterior, number of acquisition = 1160). Six additional images, three with anterior–posterior and three with posterior-anterior phase-encoding direction were collected with the same parameters in order to correct for EPI distortions^30^. MRI data of all participants was checked for incidental findings by a board certified radiologist.

### Brain parcellation

Parcellation of cortical regions and subcortical nuclei was performed using Freesurfer (Version 5.3;^31^). The cortical parcellation was based on the Desikan-Killiany atlas^32^ and the subcortical parcellation was done using the atlas implemented in Freesurfer^33^. Higher resolution cortical parcellation (234 cortical regions) was generated from the Desikan-Killiany parcellation using the connectome mapper toolbox that included the Lausanne 2008 atlas^34^. Additionally, the cerebellum was segmented in 15 subregions for each hemisphere using the rapid automatic segmentation of the human cerebellum and its lobules (RASCAL) algorithm^35^. The final parcellation used to generate the connectivity matrices included 264 brain regions (212 cortical, 22 subcortical and 30 cerebellar ROIs)^36^.

### Functional connectivity analysis

The fMRI volumes were preprocessed using FSL^37^ with the following steps: (1) motion correction; (2) distortion correction using FSL-topup^38^; (3) temporal filtering to remove physiological noise (band-pass filter = 0.01 – 0.08 Hz); (4) regression of the white matter signal, cerebrospinal fluid signal and estimated motion parameters including the outliers identified by the FSL motion outliears tool. For every MRI session and subject, the segmented T1w images were then registered to the lower resolution EPI images. For every segmented brain region, the signal of the preprocessed EPI was averaged. The FC matrices were generated by computing the cross-correlations among the averaged signals of all 264 regions. The brain regions were rearranged according to a set of canonical resting state networks as described by^39^.

### Statistical analyses

Demographic factors were compared among groups using the t-test and chi-square test. Baseline differences were analyzed using an ANOVA model with performance at BL1 or BL2 as outcome and group as the between group factor. Repetition effects between BL1 and BL2 were analyzed using linear models on the whole group level with session as within-subjects factor on neuropsychological test performance. Neuropsychological data were analyzed using a linear mixed model with test performance as outcome, the interaction term session x training group as fixed effect and study participant as random effect. χ^2^ statistics were gained by running an ANOVA over the linear model using type II sums of squares. All data were analyzed in R Studio, Version 1.2.1335^40^.

Differences in the strength of connectivity between sessions were assessed in the whole sample using the network-based statistic procedure (NBS,^41^) as implemented in the brain connectivity toolbox^42^. The procedure uses a permutation approach (number of permutations set at 10,000) to identify significant brain subnetworks with *p* value controlled for family-wise error rate (FWER < 0.05) at the subnetwork level. The statistical significance threshold testing was set at 0.05 with a t-value of four.

For groups A and B, the BL1 MRI was compared to the BL2 MRI and to the T4 MRI. For group C, the BL1 MRI was compared to the BL2 MRI and to the T3 MRI. Differences among subgroups were investigated by computing the effect size (Cohen’s *d*) of the differences of the FC strength among MRI sessions for each pair of nodes belonging to the network identified in the whole sample analysis by the NBS algorithm.

Associations between changes of connectivity strength and improvement of performance after the training were further investigated. For every subject, the average correlation coefficient of the network containing significant connections was calculated. The difference between sessions expressed in percentage was then estimated for the averaged connectivity strength and the cognitive performances. After checking for normality with the Shapiro test, a Spearman’s correlation test was conducted to study the association between connectivity changes and test performance changes.

## Results

### Behavioral results

#### Baseline characteristics of training groups

No differences were observed among groups in terms of demographic characteristics, namely sex and age (Table 1). When performing the trainings separately (Group A and B), participants did slightly more training sessions of the MSL than the WM training, independent of the order the training was given. The group that completed all trainings together, however, did approximately the same amount of sessions on both trainings (Table 1). Mean, standard deviation and range of reached levels for each training task of the different groups are displayed in Table 2.

**Table 2:** Mean (M), standard deviation (SD) and range of reached levels for WM and MSL (MOST) at the end of the training.

	WM-OP		WM-VS		WM-DT		MSL	
	M ± SD	Range	M ± SD	Range	M ± SD	Range	M ± SD	Range
Group A	22.4 ± 5.5	9–30	17 ± 4.7	10–27	17.3 ± 3.3	10–22	29.4 ± 7.2	11–38
Group B	23.6 ± 3.8	15–30	19.2 ± 5.6	12–36	18.7 ± 2.9	15–23	32.6 ± 7.7	9–42
Group C	23.9 ± 5	11–32	19.4 ± 6.9	10–41	18.1 ± 3.1	10–23	30.6 ± 7.31	15–38

OP, operation n-back task; VS, visuospatial task; DT, dual n-back task.

#### Practice effects between BL1 and BL2

The analyses indicate no differences between groups in their performance on all tasks at both the BL1 and BL2. The whole-group analyses performed to investigate possible practice effects from BL1 to BL2 resulted in a significant session effect in SDMT (χ^2^(1) = 61.63, *p* < 0.001), PASAT (χ^2^(1) = 12.92, *p* < 0.001), digit span test (χ^2^(1) = 5.21, *p* < 0.05), and 9HPT on the non-dominant left hand (χ^2^(1) = 6.69 , *p* < 0.05), with, in all cases, an increase in performance at BL2 compared to BL1 (SDMT: b = 7.07, t(53) = 7.78, *p* < 0.001, r = 0.69; PASAT: b = 2.59, t(53) = 3.56, *p* < 0.001, r = 0.44; digit span test: b = 0.70, t(53) = 2.26, *p* < 0.05, r = 0.29; 9HPT of the non-dominant left hand: b = − 0.8, t(53) =  − 2.56, *p* < 0.05, r = 0.33). Instead, there were no changes between BL1 and BL2 on the whole group level in the Corsi block tapping test (χ^2^(1) = 2.56, *p* = 0.11) and in the 9HPT of the dominant right hand (χ^2^(1) = 3.58, *p* = 0.06).

#### Training effects

The linear mixed effects models showed a significant SDMT performance improvement between sessions (χ^2^(2) = 71.09, *p* < 0.001), both at T3 compared to BL2 and T4 compared to T3. A significant session effect was found in the PASAT (χ^2^(2) = 28.26, *p* < 0.001) with an increase in performance at T3 compared to BL2 and tendency for significance between T4 compared to T3. Further, a significant digit span performance improvement between sessions (χ^2^(2) = 12.78, *p* < 0.05) was found in both at T3 compared to BL2 and at T4 compared to T3. Finally, a significant session effect was found in the 9HPT right hand (χ^2^(2) = 12.81, *p* < 0.05) with increased performance at both T3 compared to BL2 and T4 compared to T3 (Table 3). For the left hand of the 9HPT, there were no significant session, group or session by group effects. The results revealed a tendency for significance in the group by session interaction in the SDMT (χ^2^(4) = 9.01, *p* = 0.06). However, the *post-hoc* analyses yielded no significant results in the contrasts of the SDMT group by session interaction. For all other tests, neither group nor the group x session interaction was significant.

**Table 3:** Significant contrasts of linear models for session effect between BL2 versus T3 and T3 versus T4.

	BL2 versus T3					T3 versus T4				
	b	df	t	*p*	r	b	df	t	*p*	r
**Session effect**										
SDMT	− 5.76	102	− 4.76	< .001	.43	− 5.43	102	− 5.95	< .001	.51
PASAT	− 2.98	102	− 4.82	< .001	.43	− 2.11	102	− 2.33	.06	.23
Digit span test	− 1.07	102	− 3.37	< .05	.32	− 0.74	102	− 2.57	< .05	.25
9HPT right hand	0.67	102	3.14	< .05	.29	0.59	102	2.92	< .05	.28

SDMT, symbol digit modalities test; PASAT, paced auditory serial addition test; 9HPT, nine hole peg test.

The univariate analyses within group A (WM + MSL) revealed a significant improvement in performance from BL2 to T3 in the SDMT (t(17) =  − 3.09, *p* < 0.05). All other comparisons for the other tests showed no significant changes. For the group B (MSL + WM), the univariate analyses showed significant improvement within group from BL2 to T3 (t(17) =  − 3.50, *p* < 0.05) in the SDMT. The performance in the SDMT in group B further increased from T3 to T4 (t(17) =  − 3.12, *p* < 0.05) combined with an increase in the digit span performance (t(17) =  − 2.34, *p* < 0.05). In group C (MSLxWM), the univariate analyses revealed a significant increase in performance in the PASAT (t(17) =  − 2.22, *p* < 0.05) from BL2 to T3 and a significant increase in the SDMT (t(17) =  − 5.21, *p* < 0.001) from T3 to T4. All other univariate comparisons showed no significant results. All means, standard deviations, *p* values and Cohens *d* are displayed in Table 4.

**Table 4:** Means, standard deviations and univariate comparisons of the neuropsychological assessment at each session for each group.

	BL2	T3	T4	BL2 versus T3		T3 versus T4	
				*p*	d	*p*	d
**A (WM + MSL)**							
PASAT	52.17 ± 8 0.2	54.39 ± 7.52	55.94 ± 6.66	.088	.28	.144	.22
SDMT	69.17 ± 13.50	76.00 ± 17.59	78.44 ± 15.26	.007*	.39	.099	.01
Corsi Block	17.17 ± 4.25	16.89 ± 4.71	17.33 ± 4.52	.790	.06	.260	.09
Digit Span	16.56 ± 2.83	17.33 ± 3.18	17.61 ± 3.42	.181	.26	.472	.08
9HPT right	16.99 ± 1.78	16.81 ± 1.97	16.06 ± 1.77	.660	.09	.116	.34
9HPT left	17.57 ± 2.48	17.95 ± 2.67	18.05 ± 2.70	.563	.15	.854	.04
**B (MSL + WM)**							
PASAT	54.22 ± 4.31	52.00 ± 11.94	56.11 ± 5.65	.435	.24	.114	.40
SDMT	68.44 ± 14.61	75.67 ± 17.73	80.83 ± 18.20	.003*	.42	.006*	.29
Corsi Block	16.00 ± 4.39	16.89 ± 4.60	17.39 ± 4.92	.187	.19	.276	.10
Digit Span	15.67 ± 2.93	15.28 ± 3.12	16.56 ± 2.94	.537	.13	.032*	.42
9HPT right	16.72 ± 2.14	16.75 ± 1.69	16.19 ± 1.74	.948	.01	.093	.32
9HPT left	18.07 ± 1.90	18.03 ± 1.83	17.60 ± 1.31	.899	.02	.237	.26
**C (WM × MSL)**							
PASAT	53.78 ± 7.80	56.39 ± 3.99	–	.041*	.30	–	
SDMT	73.28 ± 17.79	76.50 ± 17.34	–	.127	.18	–	
Corsi Block	16.28 ± 3.56	15.39 ± 4.09	–	.311	.23	–	
Digit Span	16.67 ± 3.05	17.28 ± 3.16	–	.232	.19	–	
9HPT right	17.11 ± 1.89	16.92 ± 2.21	–	.595	.09	–	
9HPT left	17.19 ± 1.92	17.09 ± 2.11	–	.800	.05	–	

For PASAT, SDMT, corsi block and digit span number of correct answers are displayed. 9HPT was measured in seconds. **, significance level at *p* < .001, *, significance level at *p* < .05, d = Cohens’ d.

### fMRI results

**Figure 2:**
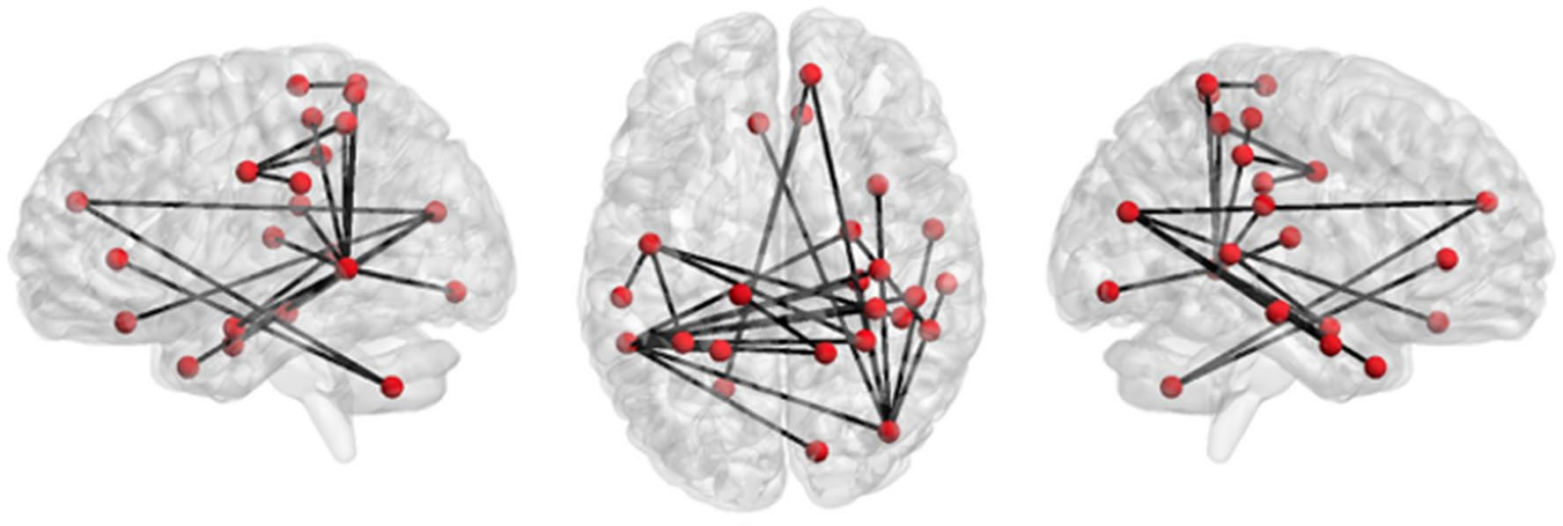
Comparison between session 1 and 4 across the whole sample. The NBS results showed increased connectivity between fronto-parietal regions in the right hemisphere and between left temporal and right parietal regions (Table 4).

**Figure 3:**
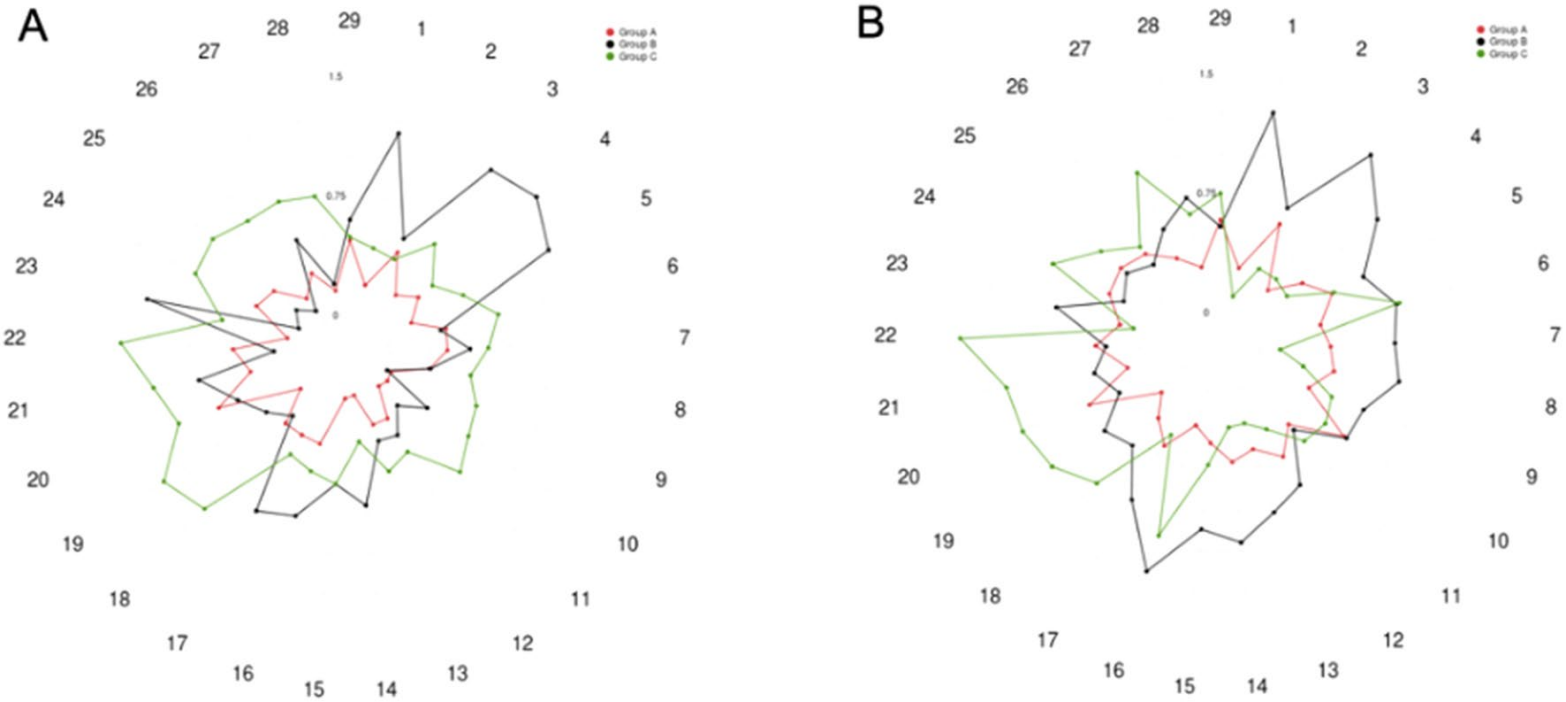
Different patterns of increased connectivity across subgroups assessed computing Cohen’s d for all significant connections in the whole sample analysis. The numbers represent the connections displayed in Table 4. Red represents group WM + MSL, black represents group MSL + WM and green represents the group WM × MSL. (**A**) session 1 versus session 3; (**B**) session 1 versus session 4.

Interestingly, the FC analysis performed on the whole sample showed no differences between the BL1 and BL2 MRI sessions. The comparisons between the BL1 (first MRI) and the T4 MRI revealed increased FC after the training in brain regions belonging to a parieto-fronto-temporal network and cerebellum, as highlighted by the NBS analysis (Fig. 2). Within this network, the right inferior parietal gyrus and the left posterior-superior temporal gyrus showed increased connectivity with the majority of the other regions suggesting increased centrality of these regions within this subnetwork (Fig. 3).

Notably, the right inferior parietal gyrus as defined in the Desikan-Killiany cortical atlas includes the angular gyrus (AG). Specifically, the right inferior parietal gyrus showed increased FC with right middle temporal gyrus, right orbitofrontal gyrus and the right hippocampus. The left posterior-superior temporal gyrus showed increased connectivity with the right superior parietal gyrus, the right supramarginal gyrus, the right postcentral gyrus, the right anterior cingulate, the right insula and right amygdala. Moreover, other relevant regions showing an increased connectivity after the trainings are the left precentral gyrus, the right superior parietal and the middle temporal gyrus bilaterally (Table 5).

**Table 5:** Connections belonging to the NBS subnetwork showing increased connectivity after training compared to BL1.

	Connections	T value	Δr
1	Right superiorfrontal 2 – right inferiorparietal	4.3	.17
2	Right inferiorparietal 6 – right temporalpole 1	4.2	.15
3	Right inferiorparietal 6 – right_middletemporal 3	4.5	.16
4	Right inferiorparietal 6 – right_middletemporal 4	4.2	.18
5	Right inferiorparietal 6 – right hippocampus	4.6	.18
6	Right bank-STS 1 – right amygdala	4.6	.17
7	Right inferiorparietal_6 – left medialorbitofrontal 2	4.4	.13
8	Right insula 1 – left parsopercularis 2	4.5	.14
9	Right hippocampus – left parsopercularis 2	4.2	.13
10	Right amygdala – left parsopercularis 2	4.9	.16
11	Right superiorparietal 2 – left precentral 1	4.1	.11
12	Right supramarginal 1 – left precentral 6	4.1	.15
13	Right superiorparietal 1 – left precentral 6	4.0	.14
14	Left precentral 6 – left supramarginal 2	4.0	.14
15	Left precentral 6 – left superiorparietal 2	4.4	.14
16	Right inferiorparietal 5 – left middletemporal 4	4.3	.14
17	Right inferiorparietal 6 – left middletemporal 4	4.2	.17
18	Right postcentral 4 – left bank-STS1	4.1	.17
19	Right postcentral 4 – left bank-STS 2	4.2	.17
20	Right supramarginal 4 – left bank-STS 2	4.5	.18
21	Right superiorparietal 1 – left bank-STS 2	4.4	.19
22	Right superiorparietal 2 – left bank-STS 2	5.0	.18
23	Right inferiorparietal 6 – left bank-STS 2	4.2	.17
24	Right lingual 3 – left bank-STS 2	4.4	.18
25	Right insula 1 – left bank-STS 2	4.8	.17
26	Right amygdala – bank-STS 2	4.3	.15
27	Left superiorparietal 1 – bank-STS 2	4.5	.17
28	Right superiorfrontal 2 – left lobuleB 7	4.1	.14
29	Right rostralanteriorcingulate 1 – left lobuleB 7	4.1	.15

Numbers next to brain region refers to the Lausanne 2008 atlas. Δr reflects the group average difference of correlation between post-training and baseline for each specific connection in the network.

The subgroup analysis performed using the Cohen’s d showed the highest effect size between BL1 and T4 in group B, and between BL1 and T3 in group C. Group B showed a Cohen’s d higher than 0.8 among regions belonging to the default mode network (DMN) and the visual network, the sensorimotor and the dorsal attention, the sensorimotor and the frontoparietal and between the limbic and visual network. The group C showed the higher effect size (> 0.8) between dorsal attention and DMN and between the sensorimotor and the DMN. Finally, group A showed the highest effect size (> 0.5) between the DMN and the dorsal attentional network, the visual network and the cerebellum (Fig. 3).

The correlation analysis between the changes in connectivity and cognitive test performance yielded no significant effects.

## Discussion

In the present study we investigated the interaction between a MSL training and a WM training in healthy subjects using brain FC and neuropsychological outcomes. On the behavioral level, increased processing speed, verbal WM and manual dexterity was found following the trainings. Although the groups did not differ in the training effects, the univariate analyses suggest that MSL conducted before WM training led to additive effects in processing speed and verbal WM. The analyses of the double BLs indicate substantial practice effects in processing speed, verbal WM and manual dexterity but not visuospatial WM. On the neurofunctional level, the analysis on the whole sample showed increased resting state FC after the trainings in a complex network that involved mainly the right angular gyrus (AG), the bank of the left superior temporal sulcus, the right superior parietal gyrus, the middle temporal gyrus bilaterally and the left precentral gyrus. The subgroup analyses showed the highest effect size for increased FC in the mentioned brain regions in the group that performed MSL before WM training, followed by the group that did both trainings combined. It is important to underline that the comparison between the two sessions performed before the trainings did not show any significant difference in FC, pointing to a training-specific effect.

All mentioned brain regions are involved in functions relevant for both MSL and WM trainings. Indeed, the AG is a region located in the portion of the inferior parietal gyrus adjacent to the temporal and occipital lobes which has been described as a continuation of the inferior parietal lobule through the superior and middle temporal gyri^43^. Due to its position, the AG is thought to have a critical role in integrating information between multiple input modalities and brain networks^43^ and has therefore been suggested to be involved in brain functions such as attention, spatial cognition, memory retrieval, reading, comprehension, number processing^44^, visuospatial attention^45^, episodic memory^46^ but also semantic memory^43,47^. Although those cognitive functions express different features, a unifying model has recently been suggested to explain the common engagement of the AG in semantic and episodic memory. The model proposes that the AG combines varying forms of information from multiple sensory modalities or spatiotemporal frameworks as an integrative dynamic buffer^48^. The integration of different inputs in the AG may then result in processes such as attention shifting to task-relevant information^43^, which could be explained through the participation of the AG in a “bottom-up” attentional subsystem^49^. Indeed, studies on FC showed that the AG is part of different networks—most consistently of the DMN, which has been associated with brain activity at rest^50^. However, recent literature suggests that the DMN – including the AG—is not only involved in rest situations, but also the unconscious processing of implicit memory^51^ and WM tasks^52^. Indeed, changes in task-related FC within hubs of the DMN, specifically in the bilateral AG have been described, which were explained through the buffer role of the AG in integrating phonological and visual processes^52^. Our study not only supports the role of the AG as an integrative region whose activity could be increased as a result of its involvement in WM and implicit memory processes, it also extends the previous studies in two ways. First, the increased FC in the AG following the trainings in our study was found at rest, which further supports the role of the AG as a DMN hub in the unconscious processing of WM. Second, the increased resting state FC in the AG showed highest effect size in the group that performed the MSL before the WM training, suggesting an involvement of the AG in motor learning.

Indeed, in our study we found an increased resting state FC in the bilateral middle temporal gyri together with the AG following the WM and MSL trainings, specifically pronounced in the MSL + WM group. The AG combined with the bilateral middle temporal gyrus have been described to be involved in action-feedback monitoring following hand movement^53^. Specifically, the angular and middle temporal gyri have been shown to be involved in intersensory conflict detection, suggesting a contribution to awareness of temporal discrepancies^53^. Furthermore, the middle temporal gyrus has also been identified in a study on healthy adults that described increased activity following simple finger movement^54^. The increased activity in the right AG, left middle frontal gyrus, bilateral post-central gyri, superior parietal gyrus and cerebellum could be defined by their role in semantic memory related to voluntary movement. The activation in the left inferior, middle and superior temporal gyri as well as the bilateral inferior frontal gyri seemed to be associated to the ideation of the finger movement and not the movement *per se*^54^*.* Indeed, previous studies described resting state FC changes in the sensorimotor and fronto-parietal networks 1 h after motor training, suggesting an offline processing of the newly learned motor skills^19^. Similarly, the middle temporal gyrus has been shown to be increased at rest following WM training^18^. Thus, the increased resting state FC in the middle temporal gyrus together with the AG in our study suggests an involvement of the middle temporal gyrus in detection and processing functions of finger movement and WM. More importantly, we detected changes in resting state which indicates an offline processing following MSL and WM training, reflecting ongoing learning mechanisms. This notion is further supported by the increased resting state FC shown in the left precentral gyrus post-training which mirrors the participants’ performance of all trainings with their dominant right hand. Additionally, resting state FC changes in the precentral gyrus following motor training have been reported previously^19,55^ which together with our findings strengthens its involvement in learning processes.

In this context, we also observed an increased FC of the right superior parietal gyrus. A large body of evidence has shown that attentional control involves the parietal cortex, including the intraparietal sulcus and superior parietal gyrus^56^. Indeed, the right superior parietal gyrus is a relevant brain region involved in sustained attention, a crucial component of learning and memory^57,58^. Additionally, it has also been reported that the bilateral superior parietal gyrus plays an important role in enhancing short-term MSL during observation of hand movements^59^. Furthermore, the increased FC in the right superior parietal gyrus found in our study was mainly related to increased FC in the left posterior-superior temporal gyrus and left superior temporal sulcus. This finding is in line with a meta-analytical connectivity model, which described a coactivation between the left superior temporal sulcus and the right superior temporal gyrus, extending to the middle temporal gyrus^60^. While the middle temporal gyrus seems to be involved in movement processes and the right superior parietal gyrus has additionally been shown to be involved in to attentional processes, the left superior temporal sulcus has been shown to be involved in the processing of visual movement consequences^61^. Overall, the described activation patterns in the superior parietal gyrus, superior temporal sulcus and middle temporal gyrus seem to correspond to the so-called dorsal stream within the dual stream theory of visuospatial processing^62,63^. According to the dual stream theory, visual information reaches the parietal lobe through the lateral intraparietal area in order to access the superior temporal sulcus and middle temporal gyrus^64^. Thus, the dorsal stream delivers information direct to the motor system for immediate use for reaching, grasping or eye movements^65^. Additionally, it has been suggested that the parieto-prefrontal pathway links the middle temporal gyrus, intraparietal areas and the prefrontal cortex, areas which have been suggested to be relevant for spatial WM^64^. By contrast, the ventral stream describing temporal regions is dedicated to ‘vision-for-perception’, but has further been described to have a role in movement planning based on memory of the object and its relationship to other items^64,65^. Despite the distinction in dorsal and ventral streams, it has been suggested that during hand movement both streams are cross-communicating through the temporo-parietal fibers^66^. The increase connectivity of the AG with other regions (middle temporal gyrus, right superior parietal gyrus, left superior temporal sulcus, left precentral gyrus) could suggest that the AG may act as an integrative region that could promote the cross-communication between dorsal and ventral streams. Additionally, they extend this notion since we observed increased FC within and between both streams in the resting brain following WM and MSL training, which can be seen as a cross-communication in terms of an offline processing.

Moreover, our results allow to understand the direction of the cross-communication, since the sequential order of the training administration modulated FC changes in the previously mentioned networks. Indeed, the post-training resting state FC changes showed the highest effect size in the group which completed the MSL first followed by the WM training and the group which did both trainings combined. Current research suggests that the motor cognitive interdependence may come from motor systems that dedicate neuronal regions to cognitive demands, which results in enhancement of active rehearsal processes through internally generated motor sequence traces that are actively recreated at will^15^. Behaviorally, we detected additive effects in visuospatial WM and processing speed following the group which first completed the MSL before the WM training. Hence, MSL training before or simultaneously with WM training seems to engage motor and cognitive brain regions which could act as a boost for WM and processing speed ability. It is important to underline that since all FC changes were detected in the brain at rest, the modulations due to the sequential order of the training seems to reflect more pronounced offline processing when the MSL is done before the WM training. The absence of FC differences between the BL1 and BL2 further supports that the observed changes in resting state FC are a result of the trainings.

The absence of correlation between cognitive and connectivity changes may be due to different reasons. First, the tasks used in the assessment are based on simple span WM paradigm compared to the tasks we used in the trainings, which are based on the n-back paradigm. Indeed, weak correlations between simple span and n-back tasks have been reported following a meta-analysis^67^. Second, the change of the performances can include also a repetition effect that is not associated with connectivity changes, as we observed comparing the two baseline measurements. Third, it has been suggested that unless the sample size is larger than 80–100 subjects, it is rather unlikely to observe reproducible brain-behavior correlations^68,69^.

Considering the relatively young age of the participants (average age 30 years) the generalization of our findings to an older population should be done with caution. Indeed, it has been described that resting state FC patterns between young and old adults differ following MSL^19^. Similarly, a recent study showed increased resting state FC following a short-term motor practice in young adults, whereas decreased FC was found in older adults^55^. For this reason, the results in this study cannot be generalized and future studies should investigate resting state FC changes following MSL and WM training in other age population, specifically old age.

To our best knowledge this is the first study that investigated the brain mechanisms at rest related to additive and interactive effects of MSL and WM trainings. The results showed distinct patterns of resting state FC modulation related to sequential and combined training programs suggesting a relevance of the order in which trainings are performed. These training-related FC modulations are supported by the absence of differences between the two pre-training sessions. Thus, the current study sheds light on the additive and interactive neuroplastic mechanisms induced by motor and cognitive trainings. Based on this observation, we think that rehabilitative programs may consider to take into account the sequential order of training administration, although further studies in pathological conditions are essential.

## Supplementary Information

Supplementary Information.
