# Supplementary material for: Additive and interaction effects of working memory and motor sequence training on brain functional connectivity
Source: Sci Rep. 2021 Nov 29;11:23089. doi: 10.1038/s41598-021-02492-9 (PMC8630199; doi:10.1038/s41598-021-02492-9)
Supplement: Supplementary file 1 — Supplementary Information. [file 41598_2021_2492_MOESM1_ESM.pdf]

Additive and interaction effects of working memory and motor sequence training on brain functional connectivity

Zuber et al.

Supplementary Material

I. Figure 1. MSL training on the tablet device.
II. Figure 2. CogniTrack WM training with A) the visuospatial, B) the operation n-back task and C) the dual-n-back task.
Table I.
III. Figure 3. Mean of maximal reached levels of participants over the 12 training sessions (days) for each group for the MSL training. Group A, WM+MSL; Group B, MSL+WM; Group C, WMxMSL.
IV. Figure 4. Mean of maximal reached levels of participants over the 12 training sessions (days) for each group. Performance in each task of the WM training is displayed. Group A, WM+MSL; Group B, MSL+WM; Group C, WMxMSL.
V. Figure 5. Mean performance of each cognitive task over the four sessions for each group.

Figure 1

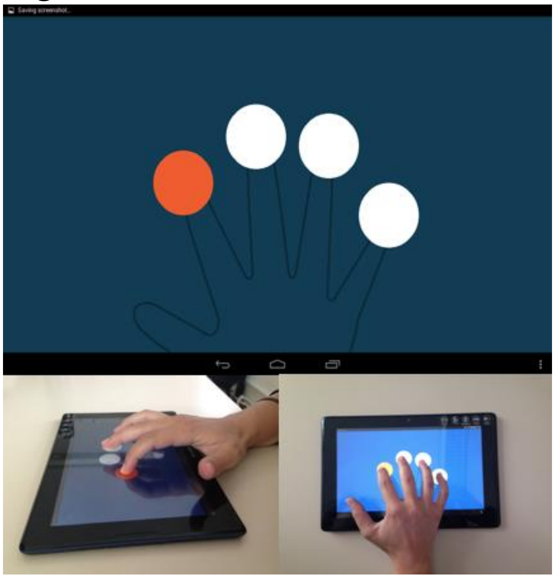

Figure 2

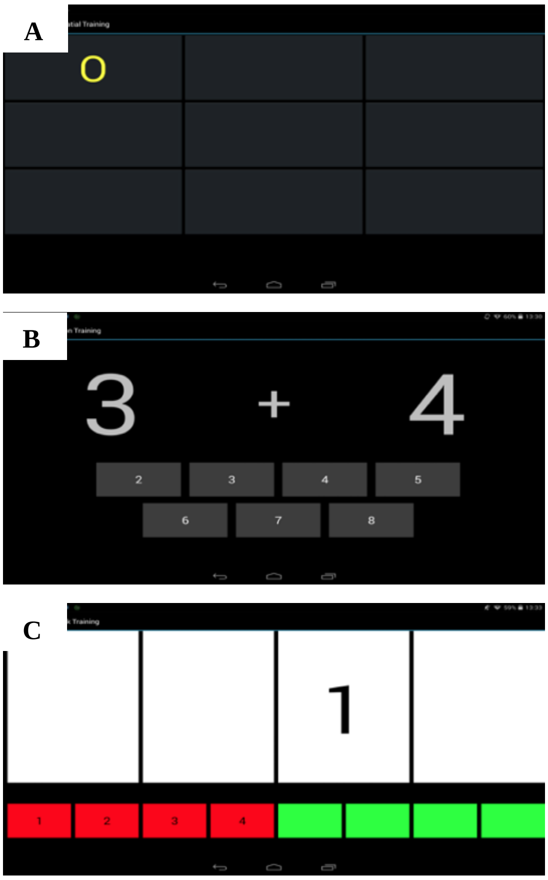

Figure 3

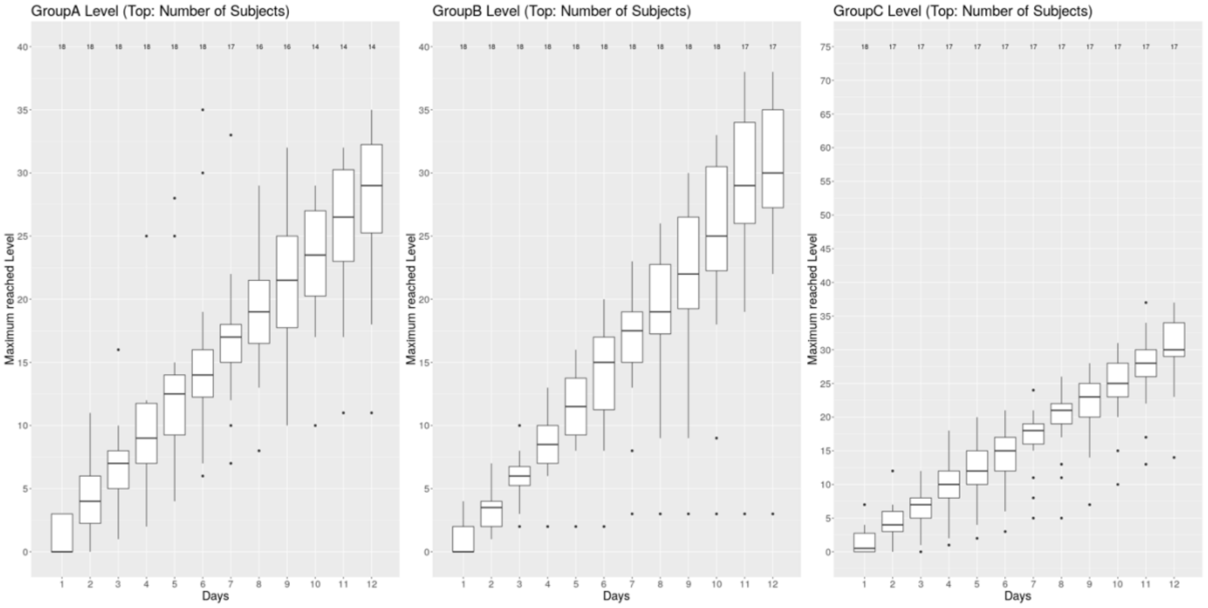

Figure 4

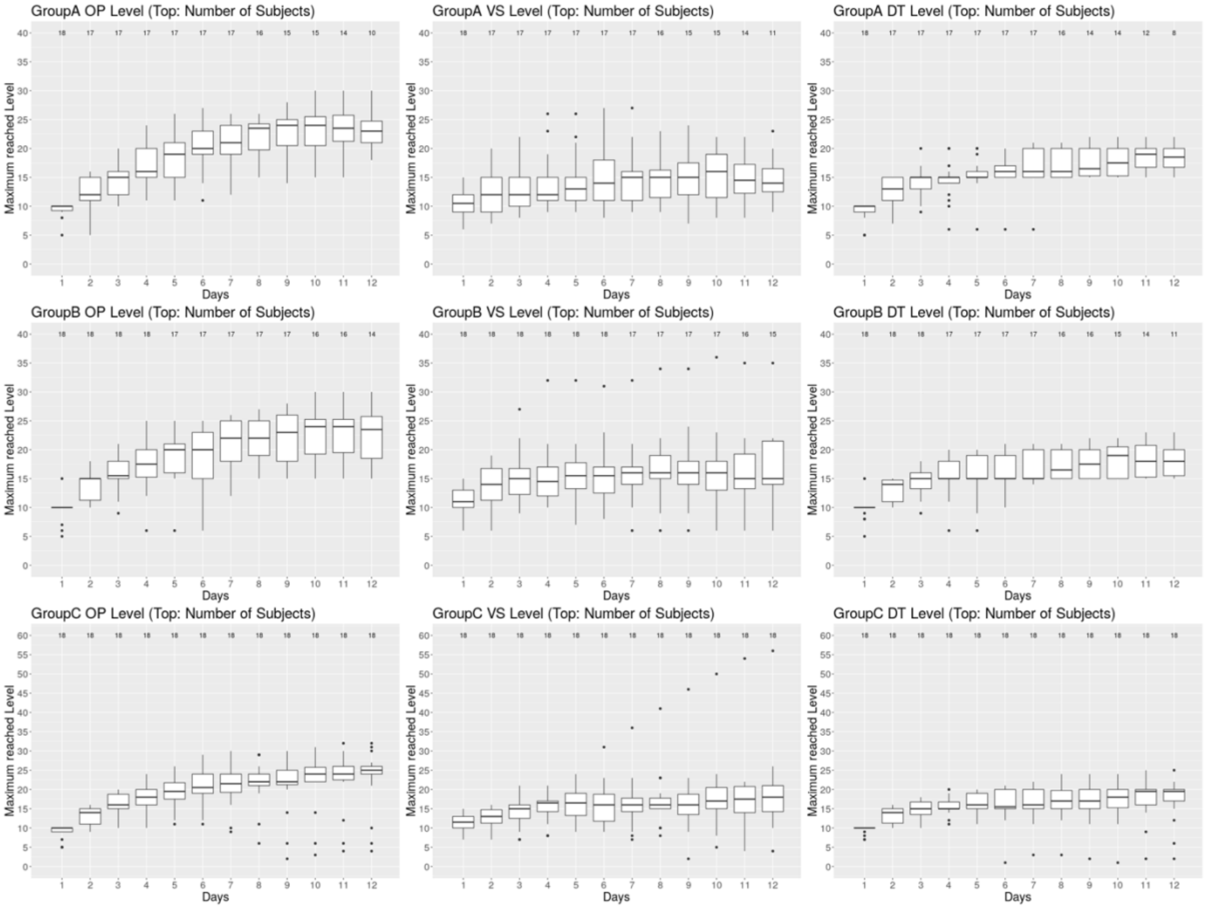

Figure 5

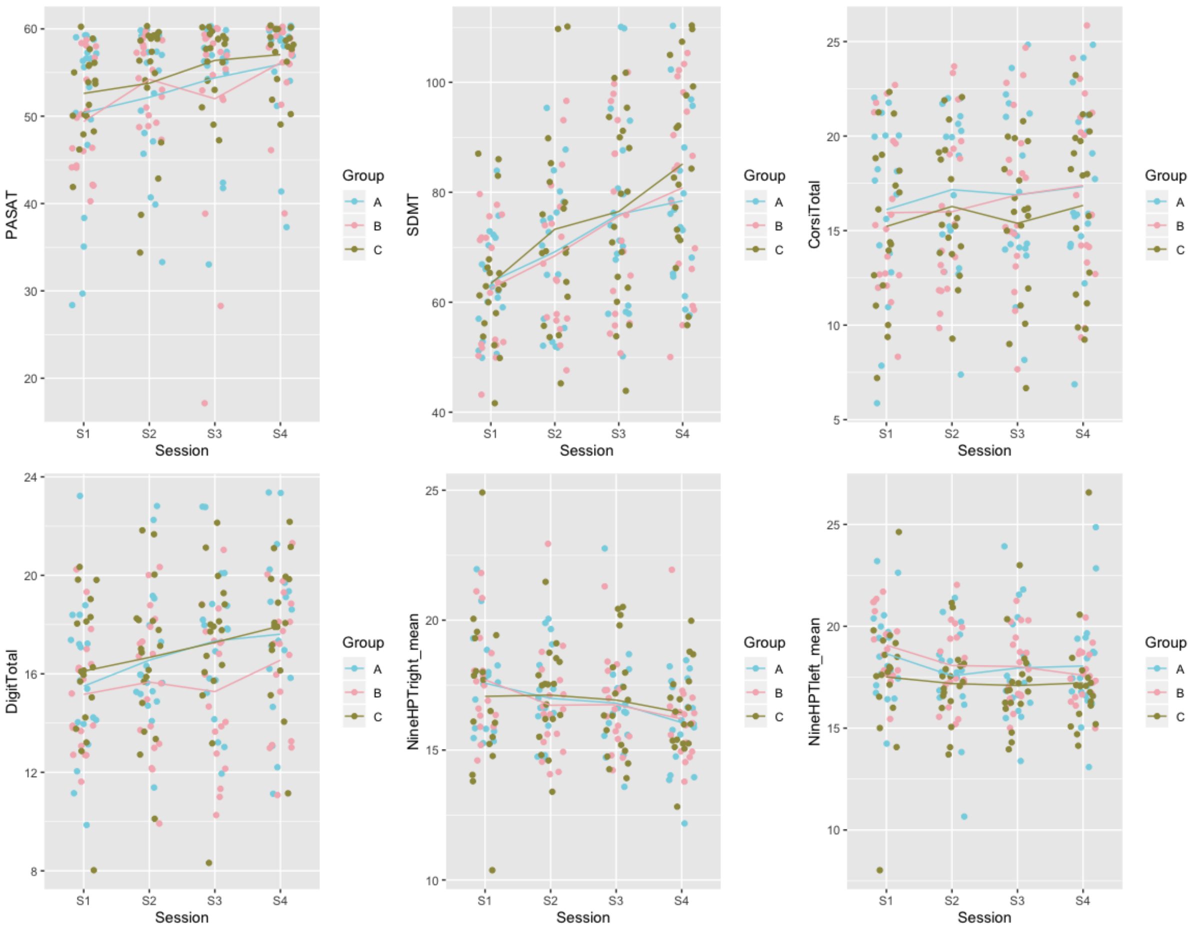
PASAT
Session
S1
S2
S3
S4
Group
A
B
C
SDMT
CorsiTotal
DigitTotal
NineHPTright_mean
NineHPTleft_mean
